# SCAN: a nanopore-based, cost effective decision-supporting tool for mass screening of aneuploidies

**DOI:** 10.1186/s40246-024-00690-w

**Published:** 2024-11-22

**Authors:** Anne Kristine Schack, M. Carmen Garrido-Navas, David Galevski, Gjorgji Madjarov, Lukasz Krych

**Affiliations:** 1 gMendel ApS, Fruebjergvej 3, 2100 Copenhagen, Denmark; 2 Food Microbiology and Fermentation, University of Copenhagen, Copenhagen, Denmark; 3 CONGEN, Genetic Counselling Services, C/Albahaca 4, Granada, Spain; 4 Facultad de Humanidades y Ciencias de la Educación, Departamento de Didáctica de las Ciencias, Universidad de Jaén, Campus Las Lagunillas s/n, Jaén, Spain; 5 Faculty of Computer Science and Engineering, University Ss Cyril and Methodius, Skopje, North Macedonia; 6 Netcetera, Zypressenstrasse 71, 8040 Zurich, Switzerland; 7 GenXone S.A., 60-476 Poznan, Poland

## Abstract

In developed countries, Newborn Screening (NBS) programs aim to detect treatable yet clinically silent disorders. The selection of disorders to be included in NBS considers severity, treatment availability, prevalence, and analysis cost. However, numerous genetic disorders remain excluded from routine testing due to high expenses and specialized equipment requirements. Here we present SCAN, a novel, non-invasive, and cost-effective decision-support tool utilizing nanopore sequencing for estimating proportions of chromosomes responsible for the most common aneuploidies. SCAN combines DNA enrichment (amplification), barcoding, nanopore sequencing, and machine learning predictive modeling. In a proof-of-concept study for Klinefelter Syndrome, SCAN achieved 100% sensitivity, specificity, and accuracy, becoming the world’s first IVD-certified genetic test utilising nanopore sequencing. Further model training shows promise in expanding this assay to detect other chromosomal aneuploidies included in the protocol.

**Supplementary Information:**

The online version contains supplementary material available at 10.1186/s40246-024-00690-w.

## Introduction

Chromosomal aneuploidy manifest in approximately 1 out of 160 live births, primarily characterized by extra copies of chromosome 21, 18, and 13 accounting for the majority of numerical autosomal alterations [1]. Sex chromosomal aneuploidies (SCAs) are also observed in a significant number, affecting 1 in 400 live births. SCAs are mostly represented by a missing or an additional X chromosome, causing Klinefelter (47, XXY) (KS) or Turner (45, X) (TS) syndromes [2].

Early detection via prenatal screening and postnatal diagnosis provides the best clinical and ethical outcomes for affected individuals. Shallow whole-genome sequencing (sWGS) in non-invasive prenatal testing (NIPT) has expanded the detection of chromosomal aneuploidies including SCAs [3–5], and raises important ethical questions regarding the appropriateness of disclosing SCAs prenatally, with some organizations, such as the European Society of Human Genetics and American Society Human Genetics, advising against routine prenatal screening for SCAs [6], while others, like the American College of Medical Genetics and Genomics, recommend it [7]. Regardless, KS remains the most undiagnosed aneuploidy.

In developed countries, autosomal aneuploidies are detected in large majority of cases, whereas SCAs remain undiagnosed more often due to the less pronounced clinical features associated with them[8–10]. Different chromosomal aneuploidies exhibit a wide range of phenotypic variations [11], from pronounced clinical features, mostly related to autosomal chromosomes to mild or no clear phenotypic signs at birth, primarily associated with SCAs. For example, KS is the most frequent sex chromosomal aneuploidy in males, yet it is estimated that only between 25 and 50% of KS is ever diagnosed [12]. The main reason for the low detection level of KS is the lack of clear clinical manifestation during infancy and childhood, often persisting until the onset of puberty [13, 14]. Furthermore, mosaic karyotype is an additional factor that reduces clinical phenotypic features, also causing problems with detection due to the reduced sensitivity of multiple tests [15–17]. Yet, many treatments are known to alleviate the symptoms related to SCAs, including hormonal, occupational, and physical therapies [18–20]. However, in order to ensure the best effectiveness of therapies, it is crucial that patients with SCAs are diagnosed early in life [14, 21–23].

**Fig. 1:**
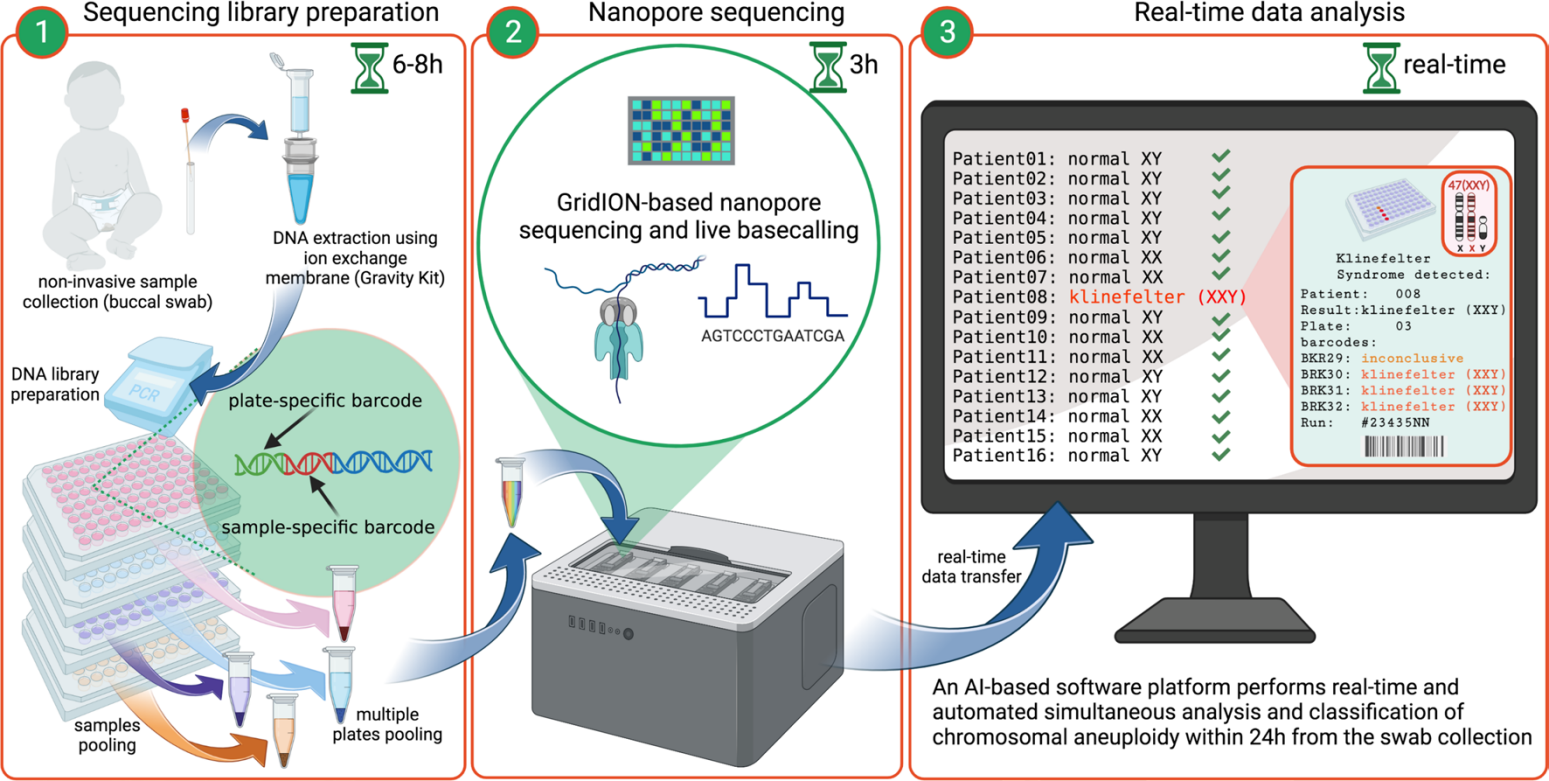
Workflow of SCAN. The workflow of the method is composed of three main steps, enabling classification of the five most common chromosomal aneuploidies in less than 24 h. **1.** Non-invasive biological material is collected for DNA extraction using a buccal swab. Samples undergo DNA extraction with an ion-exchange membrane, which ensures high molecular weight DNA. Next, samples pass through a two-step PCR protocol that facilitates non-saturated amplification of selected regions representing the chromosomes of interest and reference chromosome 15 (PCR_1_), followed by sample/technical replicate barcoding (PCR_2_). Multiple 96-well PCR plates are then pooled within each plate, and each pool receives a plate-specific barcode using the Native Barcoding Kit by Oxford Nanopore Technologies. **2.** Sequencing is performed on GridIONx5, where basecalling and first level demultiplexing (plate-specific) occur in real-time. **3.** Newly generated data is transferred to the software platform, where automated second-level demultiplexing (by sample/technical replicate) is conducted using Torchlex [24]. Read annotation is performed in real-time, enabling rapid sample classification as soon as the minimum required data is collected

To address this issue, we here present SCAN (Screening Chromosomal ANeuploidies), the first IVD-certified, decision-support tool based on Oxford Nanopore Technologies (ONT) for the postnatal detection of KS. SCAN is a fully automated, non-invasive, and cost-effective method (< $20, see Table 1 for details) that rapidly (< 24 h) evaluates the proportions of the five most common chromosomal aneuploidies (KS, TS, trisomies: 13, 18, and 21) and utilizes artificial intelligence (AI) based model to detect KS. From sample collection (buccal swab) to a decision-supported result, SCAN can sequence and analyze hundreds of samples simultaneously (Fig. 1).

**Table 1:** Single analysis costs estimations of the SCAN

Workflow’s step	Reagents	$${Cost per sample^{1}}$$
DNA extraction and quality control	Bead-Beat Micro AX Gravity kit (A&A Biotechnology, Gdansk, Poland) Qubit^®^ dsDNA HS Assay Kit (Life Technologies, Carlsbad, CA, USA)	$4.7
PCR1	PCR BIO UltraMix polymerase (PCR Biosystems Ltd, London, United Kingdom), primers, AMPure XP beads (Beckman Coulter Genomic, CA, USA)	$2.9
PCR2	PCR BIO UltraMix polymerase (PCR Biosystems Ltd, London, United Kingdom), primers/barcodes	$2.1
Library preparation	SQK-LSK110 (Oxford Naopore Technologies, Oxford, United Kindom)	$1.0
	NEBNext Ultra II End repair/dA-tailing Module (New England BioLabs, MA, USA; cat # E7546)	$0.2
	NEBNext Quick Ligation Module (New England BioLabs, MA, USA; cat # E6056)	$0.3
Quality control	Qubit^®^ dsDNA HS Assay Kit (Life Technologies, Carlsbad, CA, USA)	$0.1
Sequencing	MinION Flow Cell (Oxford Naopore Technologies, Oxford, United Kindom)	$5.7
Total cost per sample		$17.0

$^{1}$ When analyzing 24 samples simultaneously on a single MinION Flow Cell (each sample is represented in four technical replicates)

## Results

### Normalized proportions of amplicons allows for discrimination of five tested chromosomal aneuploidies

The assay development and analysis included 10 samples, of which 8 were clinically diagnosed and used to develop our platform for chromosomal classification predictions. Technical replicates of donor samples resulted in 480 samples.

**Fig. 2:**
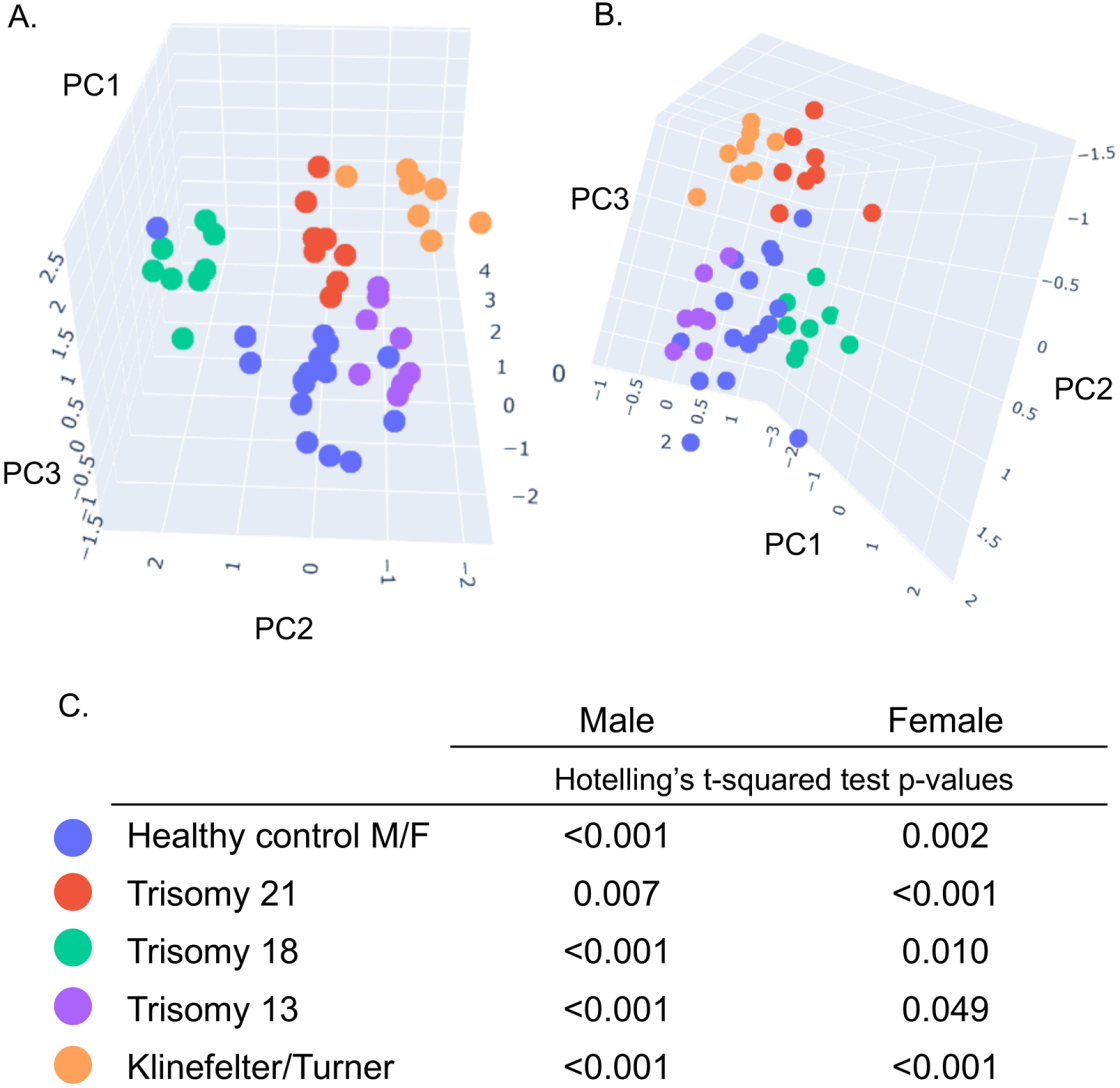
Principal Component Analysis (PCA) plots showing differential clustering of all tested chromosomal aneuploidies. Linear dimensionality reduction using Singular Value Decomposition was performed on the normalized chromosome amplicons count matrix from a “proof of concept” experiment (sample setup shown in Table S5 plate001); Three principal components were calculated for all the samples and visually presented on the PCA plots A. and B.; PCA plots showing a distinct separation between all tested aneuploidies in presence of healthy controls. Each biological sample is represented by eight technical replicates. **A.** male samples, and **B.** female samples. **C.** Hotelling’s *t*-squared test *p*-values (that measures the differences between the multivariate means of the different populations in the one vs rest setup) were calculated on the normalized chromosome amplicons count matrix

To evaluate the possibility of the method to accurately distinguish any numerical variation including (trisomies, monosomies and mosaic karyotype) from healthy controls (Table S5, plate001) a Principal Component Analysis (PCA) using Singular Value Decomposition (SVD) was performed on the normalized chromosome amplicons count matrix (reads proportions from each barcoded sample/replicate). The analysis showed significant separation between the tested aneuploidies and the controls samples in the first three components (Fig. 2A–C).

### Real-time data analysis of generated data

Current state-of-the-art ONT barcode demultiplexing tools (such as Guppy) that operate directly on the DNA base-calls are computationally expensive and their throughput is significantly lower in comparison to the existing basecalling methods. This means that they can not be applied in real-time on the stream of base-called DNA reads that are generated by the ONT device, which can significantly influence the real-time monitoring and deciding capacity about the quality and quantity of the reads per DNA sample.

The second-level demultiplexing was performed using Torchlex [24], a method for real-time demultiplexing of barcoded Oxford Nanopore reads. The method that we proposed managed to significantly reduce the computational complexity of the demultiplexing, while preserving the quality of classification compared to the competing methods. We compared its computational efficiency and predictive performance with the state-of-the-art demultiplexing method Guppy on a next-generation sequencing (NGS) run using 6 different DNA samples. The experimental validation was performed on 1,184,898 base-called DNA reads (sequence length: 900–1200bp) with a Phred quality score higher than 8 as a ground truth.

In terms of computational efficiency, the proposed method demultiplexed the base-called DNA reads by an order of magnitude faster than Guppy. The calculated throughput of Torchlex was $\sim$1520 reads/s, while the calculated throughput of Guppy was only $\sim$138 reads/s. Furthermore, it managed to significantly reduce the number of unclassified reads (6.7%) in comparison to Guppy (24%). In terms of classification performance, both methods showed very similar results. The precision and the recall of Torchlex was 97.7% and 81.4% respectively, while Guppy showed precision of 97.8% and recall of 81.3%. All the experiments were performed on one referent hardware architecture (Intel i7 10th generation, 8 cores, 32 GB RAM, no CUDA) using thread parallelism of 10.

### The analytical performance of SCAN reaches 100% for four technical replicates

The analytical performance for single technical replicates expressed with analytical sensitivity, specificity, and accuracy, scored 97%, 99.1%, and 98.6% respectively. For four technical replicates, all parameters reached 100%.

The limit of detection, defined as the proportion of 47,XXY DNA spiked with increasing concentrations of DNA from a 46,XY healthy control, demonstrated that the developed AI model can detect KS in a simulated mosaic configuration down to 25.3%. This indicates that KS can potentially be identified even when it is present in only 25.3% of the cells. This low detection limit highlights the AI model’s capability to classify KS in patients with mosaicism. Details on the performance analysis are provided in the supplementary material.

### Discussion

The diagnosis of aneuploidies has traditionally relied on labor-intensive methods such as karyotyping, fluorescence in situ hybridization (FISH), or microarray analysis although some new NGS methods can also identify copy number changes. The use of sWGS in prenatal screening has significantly improved the detection of chromosomal aneuploidies, including sex chromosome aneuploidies (SCAs [3, 4]. With sWGS data can be obtained within 24 h, and enables simultaneous analysis of a large number of samples, thus reducing the cost per sample. A number of validation experiments for these methods, such as mosaic sensitivity and resolution by NGS-based sWGS, have been described [3, 4]. Cost per sample is not define further, however, implementation of pipelines in the analytical process can increase the result efficiency and reduce cost [25]. Unlike autosomal aneuploidies, SCAs tend to be more subtle in their phenotypic expression and often present fewer immediate medical concerns [5]. As a result, many individuals with SCAs may go undiagnosed or fail to receive optimal medical care and attention throughout their lives [26]. This subtlety raises important ethical considerations when deciding whether SCAs should be included in prenatal screening. Careful thought must be given to what information is disclosed to parents, the timing of such disclosures, and how the information is communicated to avoid unnecessary distress. The American College of Medical Genetics and Genomics advocates for NIPT to be used for SCAs, emphasizing the potential benefits of early detection [7]. However, the European Society of Human Genetics and the American Society of Human Genetics do not recommend offering prenatal screening for SCAs [6]. In general, the diagnostic process for genetic disorders remains costly and time-consuming, requiring expensive specialized equipment and qualified personnel. In many cases several platforms are needed to account for their limitations in detection specific defect in DNA molecule. Currently, DNA-based molecular diagnostics are predominantly performed using Sanger sequencing for targeted sequencing and NGS (mainly Illumina), which hold necessary certifications and meet high standards in the medical sector. Despite the clear advantages of nanopore sequencing (long-read sequencing), its integration into clinical settings has been slower than expected. However, nanopore sequencing has enormous potential to transform genomics by offering longer reads, the ability to analyze methylations and structural variations, as well as native RNA. Recent advancements in nanopore sequencing technology have introduced innovative solutions, includingAI for improved quality basecalling, biological enhancements (such as new nanopores and motor proteins), and technological improvements. The read quality at a single molecule level has been a limitation of ONT compared to its main competitor in long-read sequencing, PacBio. However, this drawback has improved in recent months with the introduction of V14 chemistry, R10.4.1 Flow Cell, and updates in all protocols. In our most recent work [27], we demonstrate that ONT has finally achieved PacBio-quality reconstructions of complete bacterial genomes, but at a fraction of the cost.

Although the quality of ONT data is largely improved on the newest Flow Cells, this technology has been successfully used in clinical settings even before those upgrades. For instance, STORK, a recently developed rapid prenatal screening tool for aneuploidy in reproductive care, utilizes nanopore sequencing and represents a significant advancement in terms of universality, speed, and cost per sample [28]. Undoubtedly, the main advantage of STORK is attributed to an improved DNA extraction protocol for prenatal, invasive diagnostics and the utilization of ONT, enabling cost and time reduction in the analysis. Although STORK is limited to analyzing approximately 10 samples on a single R.9 flow cell (very likely also R10.4.1), the simultaneous analysis of a greater number of samples is necessary for mass screening [28].

The primary motivation of this study was to develop a non-invasive, rapid, and cost-effective test for postnatal screening of chromosomal aneuploidies, specifically targeting KS and TS syndromes (X chromosome aneuploidies). Early diagnosis of these syndromes can significantly improve the quality of life for affected children. Here, we present a fully validated tool for detecting KS, with ongoing work aimed at extending its application to TS in the near future (work in progress). However, our test is not limited to the X chromosome and can detect other aneuploidies as well. To train the AI model for detecting SCAs using amplicon sequencing data, our test includes signals from other chromosomes, namely 13, 18, 15 (reference signal), 21, X, and Y. The inclusion of these chromosomes is essential for the AI model to differentiate SCAs from healthy samples, as well as other possible aneuploidies. Although our data indicate that SCAN can detect trisomies 13, 18, and 21, the application of such tests is relatively low due to the clear phenotypic features present in newborns with these disorders. Consequently, we have focused on obtaining full accreditation specifically for the KS, acknowledging the complexity of the in vitro diagnostics (IVD) certification process.

KS remains the most common undiagnosed congenital condition caused by chromosomal aneuploidy [23, 29]. Despite its frequency, KS is generally not included in routine NBS programs, as immediate medical intervention has traditionally been deemed unnecessary [30]. However, KS is associated with developmental delay, behavioral problems, hypogonadism, infertility [31], and co-morbidities [32]. Studies have demonstrated that early diagnosis of KS improves patients’ quality of life and enables better medical treatment [15, 33, 34], as well as testicular sperm recovery [35, 36]. Current methods used to diagnose KS include karyotyping, chromosomal microarray analysis (CMA) and FISH [37]. Although karyotyping, CMA, and FISH demonstrate relatively high sensitivity and specificity, they all require specialized laboratory equipment and expertise to interpret the results [38]. Additionally, these methods have low throughput. The performance characteristics of karyotyping have been described with sensitivity ranging from 87 to 99% and specificity between 91 and 99.9% [39, 40]. This suggests that SCAN’s positive predictive value (PPV) is comparable to the gold standard methods for detecting KS, such as karyotyping or FISH. However, SCAN utilizes nanopore sequencing as its core technology, offering high throughput, lower infrastructure and analysis costs. Additionally, in combination with the analysis platform, it does not require highly trained personnel or involve laborious and time-consuming protocols. Lastly, SCAN offers the potential for full automation of the process, from library preparation and sequencing to data processing and decision-supporting result generation.

Nanopore technology offers three critical features not available in competing NGS platforms, which are highly valuable in molecular diagnostics and clinical settings: low-cost equipment available in various scales and throughputs, reusable flow cells, and real-time data analysis capabilities. ONT offers a broad portfolio of flow cells, providing flexibility in sample throughput, ranging from a single sample to a few dozen on a Flongle flow cell, optimal 96 samples on a GridION/MinION flow cell, and up to 2304 samples on a PromethION flow cell (24 patients, each with four technical replicates and 96 plate-specific barcodes). Additionally, ONT is currently the only technology offering real-time insight into generated data. This real-time feature is essential for the development of rapid analysis tools that can assess samples, monitor the run’s quality, and determine if sufficient data has been generated for fast decision-making. If enough data has been generated or if the data quality does not meet the required standards, the flow cell can be washed and reused, significantly impacting the cost and time of the analysis.

Thanks to the above-mentioned features, ONT, even though not yet widely adopted in diagnostic settings, offers significant advantages that in fact make it more practical for real-life scenarios. One of its key strengths is the ability to monitor data quality in real-time, allowing users to assess whether sufficient data has been generated for analysis within just a few hours of starting a run. This feature facilitates a faster turnaround time, as the run can be finalized early if enough data has been collected for decision-making. More importantly, the real-time access to data allows for rapid decision-making when handling inconclusive or failed samples that require re-analysis. Such samples can be immediately reprocessed, while other samples continue data collection. By the time sequencing is completed, re-sequenced samples experience only minimal delay, typically within a few hours. This flexibility ensures that repeated sequencing runs do not significantly impact the overall turnaround time. The capacity to isolate problematic samples for re-analysis without delaying the entire batch is a distinct advantage of ONT compared to traditional NGS platforms. Additionally, the reusability of ONT flow cells offers a high degree of flexibility, allowing for the analysis of small sample sizes without the need to pool additional samples. This stands in contrast to traditional NGS platforms, which often require larger sample pools to maintain cost-effectiveness. NGS platforms are also typically 10 to 100 times more expensive, and any errors during a run require a new flow cell, further increasing costs. Moreover, ONT’s amplicon-based protocols, such as SCAN, need only a few megabases (Mbp) of sequencing data for decision-making, whereas sWGS requires hundreds to thousands of megabases (gigabases). These attributes make ONT-based solutions like SCAN both more efficient and cost-effective for many applications.

Limitations: It is important to acknowledge the potential limitations related to the method. SCAN was validated using gDNA from patients with a clinically diagnosed aneuploidy. The extraction method and the source of samples with aneuploidy varied from those used for healthy controls. While the sample type and DNA extraction method may affect DNA concentration and purity, the relative proportions of chromosomes should remain the same across all sample types, and thus it should not significantly affect the result, provided that the quality of DNA allows amplification.

Secondly, in very rare occasions where deletion or insertion would include the region targeted by PCR, there is a possibility for the generation of false positive or false negative results. False positive results, in case of duplication, would be confronted with the confirming test. However, a false negative result could occur in the very unlikely scenario where a deletion affects the amplified region on one of the chromosomes, which could lead to a missed detection of trisomy by SCAN, despite the presence of the condition.

Lastly, SCAN was validated on a limited cohort of clinically diagnosed patients; hence, it would benefit from performance validation on a larger cohort with higher heterogeneity, including individuals with varying proportions of mosaicism.

### Conclusion

In summary, SCAN is the world’s first IVD-certified end-to-end decision support tool for non-invasive identification of Klinefelter syndrome in newborns. It serves as a proof of concept that nanopore sequencing platforms combined with AI solutions, opens up numerous opportunities for the development of rapid, low-cost, mass screening tests for a wide range of genetic disorders.

## Supplementary Information

Supplementary file 1.

## Data Availability

The raw sequencing data (fastq) is available upon reasonable request.
