# Supplementary material for: SCAN: a nanopore-based, cost effective decision-supporting tool for mass screening of aneuploidies
Source: Hum Genomics. 2024 Nov 22;18:131. doi: 10.1186/s40246-024-00690-w (PMC11583562; doi:10.1186/s40246-024-00690-w)
Supplement: Supplementary file 1 [file 40246_2024_690_MOESM1_ESM.pdf]

Supplementary materials

Methods

Sample collection, gDNA extraction and sample preparation

gDNA from patients genetically diagnosed with a chromosomal aneuploidy were purchased from Coriell Institute (Table S5). Control gDNA from healthy subjects was extracted from swab samples using Genomic Micro AX Swab Gravity Plus (A&A Biotechnology, Gdansk, Poland, 105-100P) according to the manufacturer's instructions. All gDNA samples were quantified using Qubit® 1X dsDNA HS Assay Kit on Qubit Fluorometer (Life Technologies, CA, USA). They were then normalized to approximately 1 ng/µL in UV-treated milli-q water and stored at 5°C prior to analysis. Sample stocks were stored at –80°C. The DNA extraction step is estimated to take between 2,5 to 3h for up to 24 samples.

Detection of five chromosomal aneuploidies using two-step PCR system

A two-step PCR system targets six chromosomes and incorporates patient specific barcodes. PCR_1 includes a multiplex setup of primers targeting six chromosomes containing overhangs for the second PCR (Table S6). Six specific primer pairs were designed to target chromosomes associated with the most common chromosomal aneuploidies, namely chromosomes 13, 18, 21, X, and Y. These primer pairs were optimized for high specificity to their target chromosomes while minimizing the potential for self- and hetero-dimerization. In the multiplex setup, the average sequence similarity between the generated amplicons was 37% (max = 47%, min = 32%, SD = 4%), ensuring high specificity in sequence classification. Chromosome 15 was used as a reference in the assay. The PCR_1 reaction mix contained 12µL PCRBIO UltaMix (PCR Biosystems Ltd, PA, USA PB10.32-05), 2µL of each primer set (5µM each), 1µL of DNA ($\sim$ 5ng/µL), and nuclease-free water to a total volume of 25µL. The PCR_1 thermal conditions

were optimized as follows: denaturation at 95°C for 5 min; 15 cycles at 95°C for 30 sec, 63°C for 30 sec, and 72°C for 45 sec, followed by the final elongation at 72°C for 5 min using a thermocycler (PCRmax™Alpha Cycler 1, UK). PCR_1-product was cleaned using AMPure XP Beads (Beckman Coulter Genomic, Brea, CA, USA) following manufacturer's instructions to remove excess of primers and resuspended in 11µL of nuclease-free water. The PCR_2 reaction mix included 10µL of the clean PCR_1-product, 12µL of PCRBIO UltraMix (PCR Biosystems Ltd, PA, USA PB10.32-05) and 2µL of barcode primer (10µM). The sequence of the barcodes used in PCR_2 is given in Table S7. The PCR_2 thermal conditions were as follows: 95°C for 2 min, followed by 30 cycles of 95°C for 20 sec, 55°C for 20 sec, and 72°C for 40 sec. The final extension was set to 72°C for 4 min using thermocycler (PCRmax™Alpha Cycler 1, UK). The quality control of the PCR_2-product was evaluated with gel electrophoresis on a 1,5% agarose gel stained with Midori Green (Nippon Genetics Co., Ltd., MG04, Tokyo, Japan). The two-step PCR protocol is estimated to take 2 to 2.5 h for up to 24 samples in four technical replicates.

Sequencing Libraries Preparation

PCR_2-products were pooled within each plate and cleaned with AMPure XP Beads (Beckman Coulter Genomic, Brea, CA, USA) using 250µL of the pooled PCR_2 product and 80µL of beads. The motor protein ligation was performed for each four pools individually using ligation protocol SQK-LSK110 by Oxford Nanopore Technologies (version: ACDE_9110_v110_revE_10Nov2020). The libraries were sequenced on GridION (Oxford Nanopore Technologies, Oxford, UK) using four R9.4.1 Flow Cells. The ligation protocol takes approximately 2.5 to 3 hours.

Second level Multiplexing and Sequencing with Oxford Nanopore Technologies

To test the performance of the analysis with second-level multiplexing, the four pooled libraries were subjected to barcoding using the native barcoding kit SQK-NBD112.24 by Oxford

Nanopore Technologies (version: NBA_9135_v112_revJ_01Dec2021). The final pool of 384 samples/replicates was sequenced together on a single R9.4.1 Flow Cell. The basecalling and first level demultiplexing (NBD112.24) was performed live on GridIONx5 (Oxford Nanopre Technologies, Oxford, United Kingdom) using MinKNOW GUI (version 23.03.3). The native barcoding protocol is optional when loading more than 24 samples on a single GridION flow cell and is estimated to take 3 to 3.5 hours.

Basecalling, DNA reads processing and sample and chromosome classification

The basecalling is performed using Guppy, the production basecaller integrated within MinKNOW. The signal stored within the FAST5 files is processed in real time during the run and the FASTQ files are generated one by one. Once a FASTQ file is generated, a quality check and filtering (as a first processing step) of the reads that belong to that particular FASTQ file is initiated.

Every read from the FASTQ file (one FASTQ file contains 4000 sequences) must meet certain quality criteria. The length of the sequence must be between 900 and 1200 characters and its average Phred quality score has to be higher than 8 (88% of base call accuracy). The quality score is calculated based on the average quality values of each base. If the sequence is longer than 1700 characters, the sequence is split and treated as two separate sequences. The barcode assigned to the particular sample is used as a splitting point. If the barcode is not found in the middle of the sequence (between the 100th character from left and the 100th character from right of the middle character), then the sequence is rejected and it is not used for further processing.

The processing of the qualified DNA reads continues with barcode classification or demultiplexing of the DNA sequences. In particular, the barcode that encodes the information about

the person to which the sequence read is associated should be identified. The proposed algorithm uses Levenshtein distance to find the location of the spacer (15 characters) and linker (15 characters) first, and then to identify the barcode (24 characters) located between the spacer and the linker.

The algorithm performs the search on the first and the last 150 characters of the sequence. First, we find the spacer/linker (Figure S1) by comparing a sliding window (with size 15 and stride 1) to the known values with Levenshtein Distance less than 5. The barcode should follow directly after the spacer and before the linker. Once the start and the end of the barcode are identified we find the most similar barcode from the database and perform the sample classification. The barcode search is performed using a sliding window technique with size 24 and stride 1. The distance between the window and the barcode is defined using the Levenshtein distance and it should be less than 5. This phase has low computational complexity and can be performed in real-time.

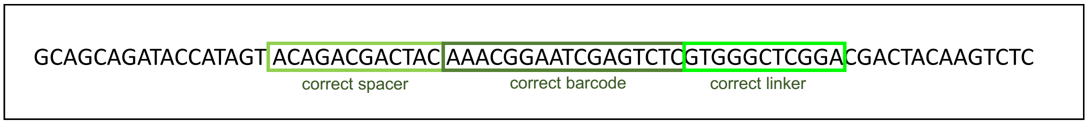

Figure S1: Correct barcode

Sometimes, the spacer or the linker could be corrupted (Figure S2), so the start and the end of the barcode could not be identified precisely. In that case we take the 24 characters that follow the spacer and try to match with the barcodes in the database. The same procedure is repeated for the 24 characters that precede the linker. The sequence that has lower Levenshtein Distance with the barcodes in the database is used for the sample classification. Again, the maximum Levenshtein Distance which is acceptable for person classification is less than 5.

To identify the end of the human DNA sequence we use the same approach. We perform the search on the last 150 characters taking into account that the linker is in opposite order with the

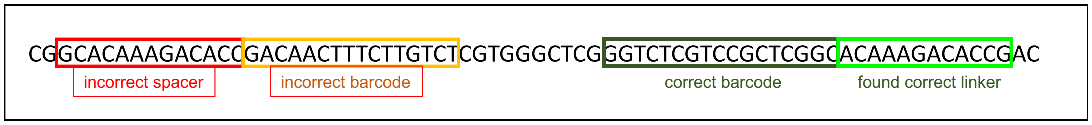

Figure S2: Corrupted barcode

replacement of characters T and G with A and C due to the reverse-complement representation.

After the demultiplexing of the sequences (sample classification), the process continues with the chromosome classification phase. In this phase, similarity scores between each demultiplexed sequence and the reference chromosome sequences are computed using the BLAST alignment method. The used scoring system awards 2 points for a base match, while penalizing 3 points for a mismatch, 3 points for extending a gap, and 5 points for opening a gap. A sequence is classified as belonging to a specific chromosome only if its final alignment score with that chromosome exceeds 250. If multiple chromosomes have alignment scores higher than 250, the sequence is assigned to the chromosome with the highest score. Sequences with alignment scores below 250 for all chromosomes are rejected and not classified. Chromosome sequences and their number grouped by barcode and chromosome are the output of this phase.

Statistical analysis

To assess whether the difference between the samples' representations from the healthy donors and the different chromosomal aneuploidies are statistically significant, a Hotelling's t-squared statistic (t^2) was performed on first three principal components. P-values showed a significant difference for all samples. Hotelling's t-squared statistic is a generalization of Student's t-statistic that is used in multivariate hypothesis testing. It tests the differences between the (multivariate) means of different populations. The test is applied on the reads proportions obtained for each barcoded sample normalized using the means and standard deviation of the controls in the one vs the rest setup (measures the difference between the population of one

disease and the combined populations from the rest of the diseases).

Performance for KS decision-supporting tool (EU IVD certification)

EU IVD certification was performed solely on the Klinefelter syndrome. The machine learning model that was built to discriminate against Klinefelter is a binary SVM classification model. First, the model was trained and tuned on a separate set of samples that included healthy, Klinefelter, Angleman (AMS), Prader–Willi syndromes (PWS) and trisomies (13, 18, and 21). After the training and the tuning phase, the model was tested and evaluated on a different set of test samples that also included cases from Klinefelter, healthy, trisomy, AMS and PWS. Details about the training and evaluation phases are given in the following subsections.

Model training and parameter tuning

In this phase, we trained a classification model and performed parameter tuning by cross validation using 278 technical replicates representing respective aneuploidy or healthy subjects (68 KS, 198 healthy, 4 trisomy syndrome, 4 AMS and 4 PWS). All the samples were obtained from 10 different individuals, generated in 5 individual runs on the Oxford Nanopore Technologies GridION x5 device. All the samples that did not meet the minimal number of reads per chromosome (100 reads per chromosome) from the 5 individual runs were filtered out and not used for training the model. In each individual run we introduced 4 male and 4 female healthy control samples, which are used to estimate the quality of the run and the variance between the different runs. All Klinefelter syndrome samples (68 technical replicates from two patients) were labeled as positive and all the other samples (including healthy and non Klinefelter syndrome samples) as negative (210). The parameter tuning of the classifier was performed using stratified 5 fold cross validation, where 80% of the data were used for training and the other 20% of the data for classifier validation. In this experiment, SVM with RBF kernel was used as a classifier. Support

Vector Machine is a natural binary classifier that works well with small to moderately sized datasets where the relationship between features and the target is not overly complex, achieving high accuracy. SVMs can handle both linear and non-linear classification tasks (by applying kernel functions like RBF, polynomial, and sigmoid, allowing it to transform the original data into a higher-dimensional space where a linear separation is possible), while avoiding the risk of overfitting, especially in scenarios with fewer data points. SVM focuses on finding the decision hyperplane that maximizes the margin between the classes, which leads to better generalization to unseen data. We have experimented with multiple different classification techniques, but the best results were achieved using the SVM classifier (in terms of predictive performance and model complexity).

The samples were represented with 8 continuous variables (features):

1. The normalized discrete probability distribution of chromosome occurrence (calculated as the proportion or percentage of identified sequences for a specific chromosome relative to the total number of sequences identified from all chromosomes in each sample) is provided for that particular sample. This is represented by columns p-chY, p-chX, p-ch21, p-ch18, p-ch13 and p-ch15 in Table S2.

2. The average Euclidean distance from the normalized discrete probability distribution of the sample to the normalized discrete probability distributions of the 4 male healthy control samples from the corresponding run (column "distance" in Table S2).

3. The ratio between the chY and chX.

The features associated with the chromosomes' representation were selected by a domain expert. Additionally, the feature that represents the average Euclidean distance between the normalized discrete probability distribution of the sample and that of the male healthy control

samples was included as a plate normalization measure. Samples closer to the healthy control distributions are more likely to be healthy compared to those farther from the control samples.

Table S1: Example of the classified reads grouped per chromosome and barcode for two KS cases

	chY	chX	ch21	ch18	ch13	ch15	total
**KS02**	4010	12936	7623	5242	1734	8047	37107
**KS91**	2048	5337	3996	3360	952	4082	19775

Table S2: Example of the dataset (after normalization and feature engineering) for the KS cases from Table S1

	p-chY	p-chX	p-ch21	p-ch18	p-ch13	p-ch15	distance	chY/chX
**KS02**	0.101	0.327	0.193	0.132	0.044	0.203	0.023	0.310
**KS91**	0.104	0.270	0.202	0.170	0.048	0.206	0.025	0.384

Model testing and performance evaluation

The evaluation of the binary SVM classifier is performed using new, unseen samples from 2 KS patients in comparison to either negative samples from two healthy donors or samples from 10 patients with other chromosomal abnormalities. Data from the healthy controls was used to calculate the analytical and the diagnostic sensitivity and data from patients with a chromosomal abnormality different from KS were used to calculate the analytical and the diagnostic specificity. Each biological sample was evaluated in quadruplicates (one different barcode for each technical replicate).

The number of true positive (TP), true negative (TN), false positive (FP) and false negative (FN) from four different sequencing runs were calculated overall for each independent technical replicate (that is, for each barcode independently) but also, per sample (that is, for each four replicates assuming that 75% of the results were coincident, either positive or negative).

Inconclusive results (either at barcode level or at patient level) were not taken into account for TP, TN, FP or FN calculations.

Klinefelter positive samples were analyzed 10 times (that is, with 40 different barcodes). Of the 40 replicates, 32 were classified by the model as ”positive” for Klinefelter, that is 80% TP, 1 was classified by as ”negative”, that is 2.5% FN and 7 were classified as ”inconclusive” (17.5%). The 32 TP allowed classifying as ”positive” 7 samples (replicates of 4) and the FN did not have an impact on analytical sensitivity as the three remaining replicates were classified as ”positive”. Three of the 10 samples were classified as inconclusive (30%).

Healthy control samples were analyzed 8 times (that is, with 32 different barcodes). Of the 32 replicates, 30 were classified by the software as ”negative” for Klinefelter, that is 94% TN, and 2 were classified by the software as ”inconclusive” (6%). These two inconclusive replicates belonged to a same patient/run, impeding to correctly classify this sample as negative (and leaving it as inconclusive); however, this did not have an impact on sensitivity or specificity calculations. Samples from the other chromosomal abnormalities were analyzed 24 times (that is, with 96 different barcodes). Of the 96 replicates, 84 were classified by the software as ”negative” for Klinefelter, that is 87.5% TN (that will be added to the 30 TN from healthy donors for the calculations); 1 was classified by the software as ”positive”, that is 1% FP and 13 were classified by the model as ”inconclusive” (all barcodes provided a results and thus all were included in the analysis). In total, there were 27 samples (either patients from other chromosomal abnormality or healthy controls) that were correctly classified as ”negative” by the system, that is 84% and 5 samples that could not be classified as they were reported as “inconclusive” (16%).

Analytical performance: Analytical sensitivity (as a measurement of precision) was assessed as the ratio of correctly positives out the total number of positives reported= TP/(TP+FP). At barcode level this value was = 32/(32+1) = 97.0%. At patient level, this value was = 7/(7+0)

Table S3: Performance parameters

	Analytical sensitivity	Analytical specificity	Accuracy
Per replicate (per barcode)	97.0%	99.1%	98.6%
Per patient (group of 4 replicates)	100%	100%	100%

= 100%.

Analytical specificity was the ratio of correctly negatives out of the total number of negatives reported = TN/(TN+FN). At barcode level this value was =114/(114+1) =99.1%. At patient level, this value was = 27/(27+0) = 100%.

Accuracy was calculated as the proportion of correct predictions using the following formula = (TP+TN)/(TP+TN+FP+FN) at both, barcode and patient levels, and data is showed in Table S3.

Clinical performance: Diagnostic sensitivity (True Positive Rate, TPR) was assessed as the number of individuals having a positive outcome of those who actually have the condition = TP/(TP+FN). At barcode level, this value was = 32/(32+1) = 97.0%. At patient level, this value was = 7/(7+0) = 100%.

Diagnostic specificity (True Negative Rate, TNR) was assessed as the number of individuals having a negative outcome of those who actually do not have the condition = TN/(TN+FP). At barcode level, this value was =114/(114+1) = 99.1%. At patient level, this value was = 27/ (27+0) = 100%.

Positive likelihood ratio (LR+) was assessed as the TPR / FPR. As value of TPR, we used the diagnostic sensitivity and as value for the FPR we used 1-diagnostic specificity = 99.1 for replicates and 0 per patient. Thus, we only could calculate LR+ for replicates = 97.0/99.1 = 0.978. Negative likelihood ratio (LR-) was assessed as FNR / TNR. As value of the FNR, we used 1-diagnostic sensitivity (3 for replicates and 0 per patient) and as value for the TNR we used the diagnostic specificity. We could only calculate LR- for replicates = 3/97.0 = 0.031.

The diagnostic odds ratio (DOR) was calculated as a ratio LR+/LR- = 0.978/0.031 =31.30.

The clinical performance described above are summarized and presented in Table S4.

Table S4: Diagnostic utility

	Diagnostic sensitivity (TPR=1-FNR)	Diagnostic specificity (TNR=1-FPR)	Positive likelihood ratio LR+=TPR/FPR	Negative likelihood ratio LR-=FNR/TNR	Diagnostic odds ratio DOR=LR+/LR-
Per replicate (per barcode)	97.0%	99.1%	0.978	0.031	32
Per patient (group of 4 replicates)	100%	100%	N/A*	N/A*	N/A*

*N/A states for not applicable as FPR for patient was 0, thus it LR+ cannot be calculated

Finally, for statistical analysis of the sensitivity/specificity, ROC analysis was done either considering the values of ”probability of being diagnosed as Klinefelter” or the outcome values of the model as follows: ”negative”=0; ”inconclusive”=1; ”positive”=2. Both analysis were performed for barcode and for sample and are visualized using the ROC curves in Figure S3

Limit of Detection: In order to evaluate the limit of detection (LOD) of the method, we simulated a KS positive sample in a mosaic configuration with decreasing ratios of 47,XXY mixed with 46,XY healthy control. The details of the assay design are in Supplementary Table 2, Plate004.

IVD certification

The device used in the study complies with the **ISO 13485:2016** and **ISO 14971:2019** standards for medical devices, as certified by **LRQA Nederland B.V.**, with Single Registration Number: **DK-MF-000018618**. The product Phivea® conforms to Directive 98/79/EC on in vitro diagnostic medical devices.

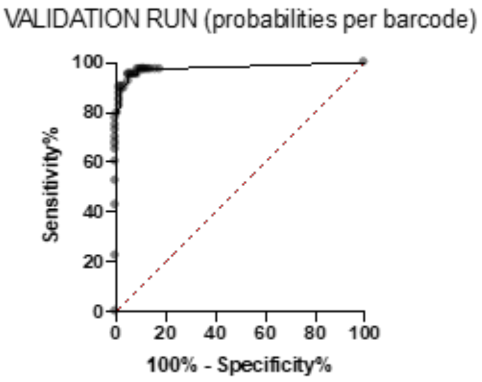

Area under the ROC curve	
Area	0.9794
Std. Error	0.01605
95% confidence interval	0.9479 to 1.000
P value	<0.0001

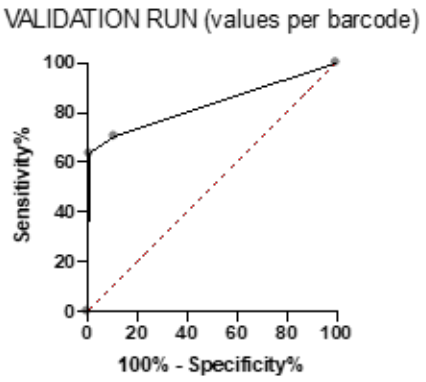

Area under the ROC curve	
Area	0.8296
Std. Error	0.04425
95% confidence interval	0.7429 to 0.9164
P value	<0.0001

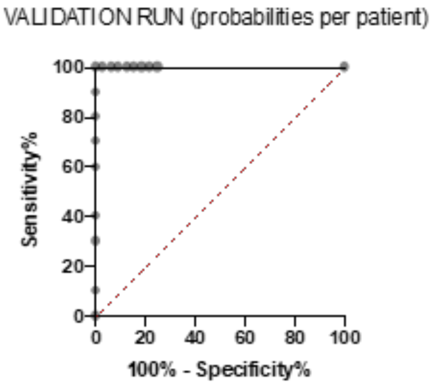

Area under the ROC curve	
Area	1.000
Std. Error	0.000
95% confidence interval	1.000 to 1.000
P value	<0.0001

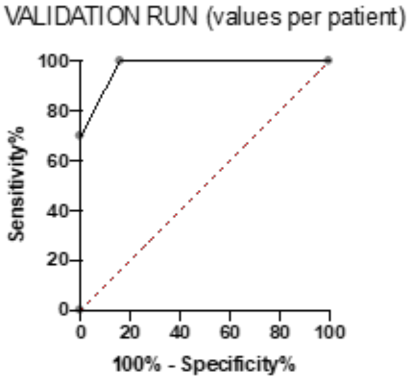

Area under the ROC curve	
Area	0.9766
Std. Error	0.01927
95% confidence interval	0.9388 to 1.000
P value	<0.0001

Figure S3: ROC curves for each barcode independently and for each patient represented by quadruplicates.

Supplementary tables

Table S5: Overview of the experimental setup and sample karyotypes

Overview of sample collection

Sample ID	KFS-02	KFS-91	TUR, M	TUR, F	TRI13, M	TRI13, F	TRI18, M	TRI18, F	PWS, M	PWS, F	AMS, M	AMS, F	TRI21, M	TRI21, F	C-M	C-F
Disorder	Klinefelter Syndrome		Turner Syndrome		Trisomy 13		Trisomy 18		Prader-Willi Syndrome		Angelman Syndrome		Trisomy 21		Healthly Controls	
Sample ID, Coriell	NA03102	NA03091	NA02668	NA10179	NA03330	NG10292	NA02732	NG13074	NA11390	NA11386	NA20375	NA11517	NA04965	NG17485	-	-
Karyotype	47,XXY	47,XXY	46,X,del(Y)(pter>q11.2:)[77]/45,X[33]	45,X	47,XY,+13	47,XX,+13	47,XY,+18	46,XX/47,XX,+18(30%/70%)	46,XY.arr(1-22,X)x2,(XY)x1	46,XX.arr(1-22,X)x2	46,XY,del(15)(pter->q11.2::q13->qter).arr[hg19]15q11.2q13.1(23641501-28560804)x1, 15q11.2(22301993-22588019)x3	46,XX.arr(1-22,X)x2	47,XY,+21	47,XX,+21	46,XY	46,XX
Experimental set-up	IVD-validation/Proof of Concept	IVD-validation	IVD-validation	IVD-validation/Proof of Concept	IVD-validation/Proof of Concept	IVD-validation/Proof of Concept	IVD-validation/Proof of Concept	IVD-validation/Proof of Concept	IVD-validation	IVD-validation	IVD-validation	IVD-validation	Proof of Concept	Proof of Concept	IVD-validation/Proof of Concept	IVD-validation/Proof of Concept

plate001, Performance of Anueploidy Segregation

Position	1 (BRK01-08)	2 (BRK09-16)	3 (BRK17-24)	4 (BRK25-32)	5 (BRK33-40)	6 (BRK41-48)	7 (BRK49-56)	8 (BRK57-64)	9 (BRK65-72)	10 (BRK73-80)	11 (BRK81-88)	12 (BRK87-96)	
A	C-M (BRK01)	C-M	C-F	C-F	TRI21, M	TRI21, F	TRI18, M	TRI18, F	TRI13, M	TRI13, F	TUR, F	KFS-02	Proof of Concept
B	C-M	C-M	C-F	C-F	TRI21, M	TRI21, F	TRI18, M	TRI18, F	TRI13, M	TRI13, F	TUR, F	KFS-02	
C	C-M	C-M	C-F	C-F	TRI21, M	TRI21, F	TRI18, M	TRI18, F	TRI13, M	TRI13, F	TUR, F	KFS-02	
D	C-M	C-M	C-F	C-F	TRI21, M	TRI21, F	TRI18, M	TRI18, F	TRI13, M	TRI13, F	TUR, F	KFS-02	
E	C-M	C-M	C-F	C-F	TRI21, M	TRI21, F	TRI18, M	TRI18, F	TRI13, M	TRI13, F	TUR, F	KFS-02	
F	C-M	C-M	C-F	C-F	TRI21, M	TRI21, F	TRI18, M	TRI18, F	TRI13, M	TRI13, F	TUR, F	KFS-02	
G	C-M	C-M	C-F	C-F	TRI21, M	TRI21, F	TRI18, M	TRI18, F	TRI13, M	TRI13, F	TUR, F	KFS-02	
H	C-M	C-M	C-F	C-F	TRI21, M	TRI21, F	TRI18, M	TRI18, F	TRI13, M	TRI13, F	TUR, F	KFS-02 (BRK96)	

plate002, Performance of segregation on selected aberrations

Position	1 (BRK01-08)	2 (BRK09-16)	3 (BRK17-24)	4 (BRK25-32)	5 (BRK33-40)	6 (BRK41-48)	7 (BRK49-56)	8 (BRK57-64)	9 (BRK65-72)	10 (BRK73-80)	11 (BRK81-88)	12 (BRK87-96)	
A	C-F (BRK01)	KFS-02	TUR, M	TRI13, F	TRI18, M	PWS, F	AMS, F	KFS-02	TUR, M	TRI13, F	TRI18, M	PWS, F	IVD-validation
B	C-F	KFS-02	TUR, M	TRI13, F	TRI18, M	PWS, F	AMS, F	KFS-02	TUR, M	TRI13, F	TRI18, M	PWS, F	
C	C-F	KFS-02	TUR, M	TRI13, F	TRI18, M	PWS, F	AMS, F	KFS-02	TUR, M	TRI13, F	TRI18, M	PWS, F	
D	C-F	KFS-02	TUR, M	TRI13, F	TRI18, M	PWS, F	AMS, F	KFS-02	TUR, M	TRI13, F	TRI18, M	PWS, F	
E	C-M	KFS-91	TUR, F	TRI13, M	TRI18, F	PWS, M	AMS, M	KFS-91	TUR, F	TRI13, M	TRI18, F	PWS, M	
F	C-M	KFS-91	TUR, F	TRI13, M	TRI18, F	PWS, M	AMS, M	KFS-91	TUR, F	TRI13, M	TRI18, F	PWS, M	
G	C-M	KFS-91	TUR, F	TRI13, M	TRI18, F	PWS, M	AMS, M	KFS-91	TUR, F	TRI13, M	TRI18, F	PWS, M	
H	C-M	KFS-91	TUR, F	TRI13, M	TRI18, F	PWS, M	AMS, M	KFS-91	TUR, F	TRI13, M	TRI18, F	PWS, M (BRK96)	

plate003, Limit of Detection | KS dilution with healthly female

Position	1 (BRK01-08)	2 (BRK09-16)	3 (BRK17-24)	4 (BRK25-32)	5 (BRK33-40)	6 (BRK41-48)	7 (BRK49-56)	8 (BRK57-64)	9 (BRK65-72)	10 (BRK73-80)	11 (BRK81-88)	12 (BRK87-96)	
A	C-F (BRK01)	AMS, F	90%-10% (KFS02-F1)	70%-30% (KFS02-F1)	50%-50% (KFS02-F1)	30%-70% (KFS02-F1)	10%-90% (KFS02-F1)	90%-10% (KFS91-F1)	70%-30% (KFS91-F1)	50%-50% (KFS91-F1)	30%-70% (KFS91-F1)	10%-90% (KFS91-F1)	IVD-validation
B	C-F	AMS, F	90%-10% (KFS02-F1)	70%-30% (KFS02-F1)	50%-50% (KFS02-F1)	30%-70% (KFS02-F1)	10%-90% (KFS02-F1)	90%-10% (KFS91-F1)	70%-30% (KFS91-F1)	50%-50% (KFS91-F1)	30%-70% (KFS91-F1)	10%-90% (KFS91-F1)	
C	C-F	AMS, F	90%-10% (KFS02-F1)	70%-30% (KFS02-F1)	50%-50% (KFS02-F1)	30%-70% (KFS02-F1)	10%-90% (KFS02-F1)	90%-10% (KFS91-F1)	70%-30% (KFS91-F1)	50%-50% (KFS91-F1)	30%-70% (KFS91-F1)	10%-90% (KFS91-F1)	
D	C-F	AMS, F	90%-10% (KFS02-F1)	70%-30% (KFS02-F1)	50%-50% (KFS02-F1)	30%-70% (KFS02-F1)	10%-90% (KFS02-F1)	90%-10% (KFS91-F1)	70%-30% (KFS91-F1)	50%-50% (KFS91-F1)	30%-70% (KFS91-F1)	10%-90% (KFS91-F1)	
E	C-M	AMS, M	80%-20% (KFS02-F1)	60%-40% (KFS02-F1)	40%-60% (KFS02-F1)	20%-80% (KFS02-F1)	KFS-02	80%-20% (KFS91-F1)	60%-40% (KFS91-F1)	40%-60% (KFS91-F1)	20%-80% (KFS91-F1)	KFS-91	
F	C-M	AMS, M	80%-20% (KFS02-F1)	60%-40% (KFS02-F1)	40%-60% (KFS02-F1)	20%-80% (KFS02-F1)	KFS-02	80%-20% (KFS91-F1)	60%-40% (KFS91-F1)	40%-60% (KFS91-F1)	20%-80% (KFS91-F1)	KFS-91	
G	C-M	AMS, M	80%-20% (KFS02-F1)	60%-40% (KFS02-F1)	40%-60% (KFS02-F1)	20%-80% (KFS02-F1)	KFS-02	80%-20% (KFS91-F1)	60%-40% (KFS91-F1)	40%-60% (KFS91-F1)	20%-80% (KFS91-F1)	KFS-91	
H	C-M	AMS, M	80%-20% (KFS02-F1)	60%-40% (KFS02-F1)	40%-60% (KFS02-F1)	20%-80% (KFS02-F1)	KFS-02	80%-20% (KFS91-F1)	60%-40% (KFS91-F1)	40%-60% (KFS91-F1)	20%-80% (KFS91-F1)	KFS-91 (BRK96)	

plate004, Limit of Detection | Performance of simulation of KS Mosaicism Detection with healthly male and Minimum DNA concentration recognised for detection

Position	1 (BRK01-08)	2 (BRK09-16)	3 (BRK17-24)	4 (BRK25-32)	5 (BRK33-40)	6 (BRK41-48)	7 (BRK49-56)	8 (BRK57-64)	9 (BRK65-72)	10 (BRK73-80)	11 (BRK81-88)	12 (BRK87-96)	
A	C-F (BRK01)	90%-10% (KFS02-M1)	70%-30% (KFS02-M1)	50%-50% (KFS02-M1)	30%-70% (KFS02-M1)	10%-90% (KFS02-M1)	90%-10% (KFS91-M1)	70%-30% (KFS91-M1)	50%-50% (KFS91-M1)	30%-70% (KFS91-M1)	10%-90% (KFS91-M1)	KFS-02, 9:1	IVD-validation
B	C-F	90%-10% (KFS02-M1)	70%-30% (KFS02-M1)	50%-50% (KFS02-M1)	30%-70% (KFS02-M1)	10%-90% (KFS02-M1)	90%-10% (KFS91-M1)	70%-30% (KFS91-M1)	50%-50% (KFS91-M1)	30%-70% (KFS91-M1)	10%-90% (KFS91-M1)	KFS-02, 9:1	
C	C-F	90%-10% (KFS02-M1)	70%-30% (KFS02-M1)	50%-50% (KFS02-M1)	30%-70% (KFS02-M1)	10%-90% (KFS02-M1)	90%-10% (KFS91-M1)	70%-30% (KFS91-M1)	50%-50% (KFS91-M1)	30%-70% (KFS91-M1)	10%-90% (KFS91-M1)	KFS-02, 9:1	
D	C-F	90%-10% (KFS02-M1)	70%-30% (KFS02-M1)	50%-50% (KFS02-M1)	30%-70% (KFS02-M1)	10%-90% (KFS02-M1)	90%-10% (KFS91-M1)	70%-30% (KFS91-M1)	50%-50% (KFS91-M1)	30%-70% (KFS91-M1)	10%-90% (KFS91-M1)	KFS-02, 9:1	
E	C-M	80%-20% (KFS02-M1)	60%-40% (KFS02-M1)	40%-60% (KFS02-M1)	20%-80% (KFS02-M1)	KFS-02	80%-20% (KFS91-M1)	60%-40% (KFS91-M1)	40%-60% (KFS91-M1)	20%-80% (KFS91-M1)	KFS-91	KFS-91, 9:1	
F	C-M	80%-20% (KFS02-M1)	60%-40% (KFS02-M1)	40%-60% (KFS02-M1)	20%-80% (KFS02-M1)	KFS-02	80%-20% (KFS91-M1)	60%-40% (KFS91-M1)	40%-60% (KFS91-M1)	20%-80% (KFS91-M1)	KFS-91	KFS-91. 9:1	
G	C-M	80%-20% (KFS02-M1)	60%-40% (KFS02-M1)	40%-60% (KFS02-M1)	20%-80% (KFS02-M1)	KFS-02	80%-20% (KFS91-M1)	60%-40% (KFS91-M1)	40%-60% (KFS91-M1)	20%-80% (KFS91-M1)	KFS-91	KFS-91, 9:1	
H	C-M	80%-20% (KFS02-M1)	60%-40% (KFS02-M1)	40%-60% (KFS02-M1)	20%-80% (KFS02-M1)	KFS-02	80%-20% (KFS91-M1)	60%-40% (KFS91-M1)	40%-60% (KFS91-M1)	20%-80% (KFS91-M1)	KFS-91	KFS-91, 9:1 (BRK96)	

plate005, Limit of Detection | Minimum DNA concentration recognised for detection

Position	1 (BRK01-08)	2 (BRK09-16)	3 (BRK17-24)	4 (BRK25-32)	5 (BRK33-40)	6 (BRK41-48)	7 (BRK49-56)	8 (BRK57-64)	9 (BRK65-72)	10 (BRK73-80)	11 (BRK81-88)	12 (BRK87-96)	
A	C-F (BRK01)	KFS-02, 8:2	KFS-02, 7:3	KFS-02, 6:4	KFS-02, 5:5	KFS-02, 4:6	KFS-02, 3:7	KFS-02, 2:8	KFS-02, 1:9	TUR, M	TRI13, F	TRI18, M	IVD-validation
B	C-F	KFS-02, 8:2	KFS-02, 7:3	KFS-02, 6:4	KFS-02, 5:5	KFS-02, 4:6	KFS-02, 3:7	KFS-02, 2:8	KFS-02, 1:9	TUR, M	TRI13, F	TRI18, M	
C	C-F	KFS-02, 8:2	KFS-02, 7:3	KFS-02, 6:4	KFS-02, 5:5	KFS-02, 4:6	KFS-02, 3:7	KFS-02, 2:8	KFS-02, 1:9	TUR, M	TRI13, F	TRI18, M	
D	C-F	KFS-02, 8:2	KFS-02, 7:3	KFS-02, 6:4	KFS-02, 5:5	KFS-02, 4:6	KFS-02, 3:7	KFS-02, 2:8	KFS-02, 1:9	TUR, M	TRI13, F	TRI18, M	
E	C-M	KFS-91, 8:2	KFS-91, 7:3	KFS-91, 6:4	KFS-91, 5:5	KFS-91, 4:6	KFS-91, 3:7	KFS-91, 2:8	KFS-91. 1:9	KFS-02	KFS-91	TUR, F	
F	C-M	KFS-91. 8:2	KFS-91, 7:3	KFS-91, 6:4	KFS-91. 5:5	KFS-91, 4:6	KFS-91, 3:7	KFS-91, 2:8	KFS-91, 1:9	KFS-02	KFS-91	TUR, F	
G	C-M	KFS-91, 8:2	KFS-91, 7:3	KFS-91, 6:4	KFS-91, 5:5	KFS-91, 4:6	KFS-91, 3:7	KFS-91, 2:8	KFS-91, 1:9	KFS-02	KFS-91	TUR, F	
H	C-M	KFS-91. 8:2	KFS-91, 7:3	KFS-91, 6:4	KFS-91. 5:5	KFS-91, 4:6	KFS-91, 3:7	KFS-91, 2:8	KFS-91. 1:9	KFS-02	KFS-91	TUR, F (BRK96)	

Plate00X represents the experimental setup on a standard 96-well PCR plate, indicated by rows (A-H) and columns (1-12). Columns 1-12 contain technical replicates of four samples, each assigned a unique barcode (BRK).

Table S6: Primers sequence for the multiplex setup. Sequence marked in bold represents the overhang which is a target for barcoding primers during PCR_2

**Chromosome Y**	
Forward	**5'-GTCTCGTGGGCTCGG**GATCTCTTCAGCGTGGGAGG-'3
Revers	3'-**GTCTCGTGGGCTCGG**CTGAGAGGGTGAGGACAGGA-'5
**Chromosome X**	
Forward	**5'-GTCTCGTGGGCTCGG**GAGAGGGAGGAACGCATAGC-'3
Revers	3'-**GTCTCGTGGGCTCGG**AGGGAGCAGAATTGAGGCAC-'5
**Chromosome 21**	
Forward	5'-GTCTCGTGGGCTCGGGTCCCCAGGTAACATCCACG-'3
Revers	3'-GTCTCGTGGGCTCGGAGCTATAAGCCAAGGGACGC-'5
**Chromosome 18**	
Forward	**5'-GTCTCGTGGGCTCGG**GTCTAGAGTGGGAGGGGCTA-'3
Revers	3'-**GTCTCGTGGGCTCGG**CCAGCTGGCTGAAATGAAGAA-'5
**Chromosome 13**	
Forward	**5'-GTCTCGTGGGCTCGG**TAGTTGAGGGGTGGCTTTGC-'3
Revers	3'-**GTCTCGTGGGCTCGG**CCTACCTCCCTGTTCTCTGC-'5
**Chromosome 15**	
Forward	**5'-GTCTCGTGGGCTCGG**GTGGGCAGCTTCCAGTAGTT-'3
Revers	3'-**GTCTCGTGGGCTCGG**CTGAGACTCGGGGTTTGCAT-'5

PCR_1 targets the gene of interest during unsaturated PCR and incorporates "linker" sequence that will become a primer binding site for PCR_2. The linker sequence is palindromic, hence only one barcode primer is needed in PCR_2. Each primer includes 15 bp spacer separating ONT motor protein adapter from the barcode sequence. The spacer was added to ensure higher tolerance for the low quality at the beginning of the sequence entering the pore and thus higher recovery of barcode sequence. The barcoded DNA constructs are subsequently pooled and motor protein is added using the ligation protocol.

Table S7: Sequence of oligonucleotides used for barcoding with PCR_2

barcode name	spacer	barcode sequence	linker
BRK01	GTCTCGTCCGCTCGG	CACAAAGACACCGACAACTTTCTT	GTCTCGTGGGCTCGG
BRK02	GTTAGTTGATGTAGT	ACAGACGACTACAAACGGAATCGA	GTCTCGTGGGCTCGG
BRK03	GTCTCGTCCGCTCGG	CCTGGTAACTGGGACACAAGACTC	GTCTCGTGGGCTCGG
BRK04	GTTAGTTGATGTAGT	TAGGGAAACACGATAGAATCCGAA	GTCTCGTGGGCTCGG
BRK05	GTCTCGTCCGCTCGG	AAGGTTACACAAACCCTGGACAAG	GTCTCGTGGGCTCGG
BRK06	GTTAGTTGATGTAGT	GACTACTTTCTGCCTTTGCGAGAA	GTCTCGTGGGCTCGG
BRK07	GATATGATATAGATA	AAGGATTCATTCCCACGGTAACAC	GTCTCGTGGGCTCGG
BRK08	GTTAGTTGATGTAGT	ACGTAACTTGGTTTGTTCCCTGAA	GTCTCGTGGGCTCGG
BRK09	GTCTCGTCCGCTCGG	AACCAAGACTCGCTGTGCCTAGTT'	GTCTCGTGGGCTCGG
BRK10	GTTAGTTGATGTAGT	GAGAGGACAAAGGTTTCAACGCTT	GTCTCGTGGGCTCGG
BRK11	GTTAGTTGATGTAGT	TCCATTCCCTCCGATAGATGAAAC	GTCTCGTGGGCTCGG
BRK12	GTCTCGTCCGCTCGG	TCCGATTCTGCTTCTTTCTACCTG	GTCTCGTGGGCTCGG
BRK13	GTCTCGTCCGCTCGG	TCACACGAGTATGGAAGTCGTTCT	GTCTCGTGGGCTCGG
BRK14	TACATTGATGCATGG	TCTATGGGTCCCAAGAGACTCGTT	GTCTCGTGGGCTCGG
BRK15	GTTAGTTGATGTAGT	CAGTGGTGTTAGCGAGGTAGACCT	GTCTCGTGGGCTCGG
BRK16	TACATTGATGCATGG	AGTACGAACCACTGTCAGTTGACG	GTCTCGTGGGCTCGG
BRK17	GTCTCGTCCGCTCGG	ATCAGAGGTACTTTCCTGGAGGGT	GTCTCGTGGGCTCGG
BRK18	GTTAGTTGATGTAGT	GCCTATCTAGGTTGTTGGGTTTGG	GTCTCGTGGGCTCGG
BRK19	GTTAGTTGATGTAGT	ATCTCTTGACACTGCACGAGGAAC	GTCTCGTGGGCTCGG
BRK20	GTTAGTTGATGTAGT	ATGAGTTCTCGTAACAGGACGCAA	GTCTCGTGGGCTCGG
BRK21	GTTAGTTGATGTAGT	TAGAGAACGGACAATGAGAGGCTC	GTCTCGTGGGCTCGG
BRK22	GTTAGTTGATGTAGT	CGTACTTTGATACATGGCAGTGGT	GTCTCGTGGGCTCGG
BRK23	GTCTCGTCCGCTCGG	CGAGGAGGTTCACTGGGTAGTAAG	GTCTCGTGGGCTCGG
BRK24	GTTAGTTGATGTAGT	CTAACCCATCATGCAGAACTATGC	GTCTCGTGGGCTCGG
BRK25	GTCTCGTCCGCTCGG	CATTGCGTTGCATACCCAACTTAC	GTCTCGTGGGCTCGG
BRK26	TACATTGATGCATGG	ATGAGAATGCGTAGTCGCTGTATG	GTCTCGTGGGCTCGG
BRK27	GTCTCGTCCGCTCGG	TGTAAGAGGTGAATCTAACCGTCG	GTCTCGTGGGCTCGG
BRK28	GTTAGTTGATGTAGT	GATACGGTGCCTTCTTAGGTTTCA	GTCTCGTGGGCTCGG
BRK29	GTTAGTTGATGTAGT	GGTCTGTCAACCCAAGGTGTCTAG	GTCTCGTGGGCTCGG
BRK30	GTTAGTTGATGTAGT	TGGGTCGAAGTAGATCCTCACTGA	GTCTCGTGGGCTCGG
BRK31	GTCTCGTCCGCTCGG	CAATGTAACTGATTGCTGTACGCA	GTCTCGTGGGCTCGG
BRK32	GTTAGTTGATGTAGT	ATGACGTTGTCGGACTTCTACTGG	GTCTCGTGGGCTCGG
BRK33	GTCTCGTCCGCTCGG	AGTTACCCAACCGTACCAAGTCTG	GTCTCGTGGGCTCGG
BRK34	GTTAGTTGATGTAGT	GCCTTTGACTTGAGTTCTTCGTCC	GTCTCGTGGGCTCGG
BRK35	GTCTCGTCCGCTCGG	GCAGTCCCTCAGCTTCGTAAGTAG	GTCTCGTGGGCTCGG
BRK36	GTTAGTTGATGTAGT	TGTTTCCTCCTCTAACTGGGACAT	GTCTCGTGGGCTCGG
BRK37	GTCTCGTCCGCTCGG	TGATACTAAGCATCAATCGCAAGC	GTCTCGTGGGCTCGG
BRK38	GTTAGTTGATGTAGT	TTCTCTGTATCGTCCTCCTGTGGT	GTCTCGTGGGCTCGG
BRK39	GTTAGTTGATGTAGT	GAGAGGCTCTAGTTGACACTGTGG	GTCTCGTGGGCTCGG
BRK40	GTTAGTTGATGTAGT	GGCTATCCTTGGTCATCCAAACTA	GTCTCGTGGGCTCGG
BRK41	GTTAGTTGATGTAGT	CGTGTACTTCTCTGGACGAACTCC	GTCTCGTGGGCTCGG
BRK42	GTCTCGTCCGCTCGG	CTGGCAGGTATGCCTTACACGTAG	GTCTCGTGGGCTCGG
BRK43	GTTAGTTGATGTAGT	CTACCGTCGAGTCAACAACGAAAG	GTCTCGTGGGCTCGG
BRK44	GTTAGTTGATGTAGT	GAGTGGGAAGGAACCCTTTCTACT	GTCTCGTGGGCTCGG
BRK45	GTCTCGTCCGCTCGG	CACTGAAGGCATCTCTGTTGGATC	GTCTCGTGGGCTCGG
BRK46	GTTAGTTGATGTAGT	CAGGAGAATGAAGTGGAACACAGC	GTCTCGTGGGCTCGG
BRK47	GTCTCGTCCGCTCGG	GAACTACCTGTGGGAAAGTTGCAC	GTCTCGTGGGCTCGG
BRK48	GTTAGTTGATGTAGT	TACAGGTGTACCACGTTCCAGATG	GTCTCGTGGGCTCGG
BRK49	GTCTCGTCCGCTCGG	CTAGATGTTCAAAGCTGCACCAGT	GTCTCGTGGGCTCGG
BRK50	GTTAGTTGATGTAGT	ACGCAGGAAGTTACCAAAGTCCAT	GTCTCGTGGGCTCGG
BRK51	GTCTCGTCCGCTCGG	GAGGACCCAGTAGGCTCATTCAAC	GTCTCGTGGGCTCGG
BRK52	TACATTGATGCATGG	GTCCACGAACAATCTTGTCTCTCA	GTCTCGTGGGCTCGG
BRK53	GTCTCGTCCGCTCGG	CTTTGCATGAGACGGTCTGAATCT	GTCTCGTGGGCTCGG
BRK54	GTTAGTTGATGTAGT	CATGCTCCTTAGTCAAAGCTCTTG	GTCTCGTGGGCTCGG
BRK55	GTCTCGTCCGCTCGG	CGTAGATCAGGGTCTCATCTTCCA	GTCTCGTGGGCTCGG
BRK56	GTCTCGTCCGCTCGG	TTCATGCCACCTGTTGAGTAGTGA	GTCTCGTGGGCTCGG
BRK57	TACATTGATGCATGG	ACTTCCGAAGGAGATTGACCTAGC	GTCTCGTGGGCTCGG
BRK58	GTTAGTTGATGTAGT	TCAGACTCACGGAGGAGTAACCTG	GTCTCGTGGGCTCGG
BRK59	GTTAGTTGATGTAGT	ACCTTGCTTTCCCTTCTTGATTGA	GTCTCGTGGGCTCGG
BRK60	GTTAGTTGATGTAGT	CCATAGAAGCCTTGGTTGAACATG	GTCTCGTGGGCTCGG
BRK61	TACATTGATGCATGG	GTGCTGAGGCACATAGTACCCTCT	GTCTCGTGGGCTCGG
BRK62	GTTAGTTGATGTAGT	TACGTCCTGAAGTAAGTGTGGGTG	GTCTCGTGGGCTCGG
BRK63	GTTAGTTGATGTAGT	GTTCAAGACCCAGGAACTTCAGAA	GTCTCGTGGGCTCGG
BRK64	GTTAGTTGATGTAGT	GAAAGTCGATGAACGGTGTCTGTC	GTCTCGTGGGCTCGG
BRK65	GTCTCGTCCGCTCGG	CCTTGTCTGGAGGAAGACTGAGAA	GTCTCGTGGGCTCGG
BRK66	GTCTCGTCCGCTCGG	GAAGTTAGAAGCCACAAGGATCGG	GTCTCGTGGGCTCGG
BRK67	TACATTGATGCATGG	GGTGAGCACACGAGTATGACAAAC	GTCTCGTGGGCTCGG
BRK68	GTCTCGTCCGCTCGG	CCACCTTCGTGTTTGCTTAGATTC	GTCTCGTGGGCTCGG
BRK69	GTTAGTTGATGTAGT	AGATCACATGAGGCTCGGACTGTA	GTCTCGTGGGCTCGG
BRK70	GTTAGTTGATGTAGT	ACACTCCATTCGTAGGATCTCGGT	GTCTCGTGGGCTCGG
BRK71	GTCTCGTCCGCTCGG	CTGTTACTACCTGATGCTCCCAGG	GTCTCGTGGGCTCGG
BRK72	GTTAGTTGATGTAGT	GTCGGTATGGAAGACAGTCAGCTA	GTCTCGTGGGCTCGG
BRK73	GTCTCGTCCGCTCGG	GAGGGTTCTGTCATCCTGTTTCTT	GTCTCGTGGGCTCGG
BRK74	GTTAGTTGATGTAGT	AGTGGAAGTGTTGGGATGCTTGTA	GTCTCGTGGGCTCGG
BRK75	GTCTCGTCCGCTCGG	ACAACAGGGTTCATCACAATGGTC	GTCTCGTGGGCTCGG
BRK76	GTTAGTTGATGTAGT	GTCCAGGGTTGATGTAACAAGCAT	GTCTCGTGGGCTCGG
BRK77	GTCTCGTCCGCTCGG	GTTGTATCCCTGAGAAACAGGTCG	GTCTCGTGGGCTCGG
BRK78	GTTAGTTGATGTAGT	TTCTGATTCAAAGGTTCGGTTGTT	GTCTCGTGGGCTCGG
BRK79	GTCTCGTCCGCTCGG	CAGCAGTGAGAACTATCTCCGAGA	GTCTCGTGGGCTCGG
BRK80	GTTAGTTGATGTAGT	GAATCGCTATCCTATGTTCATCCG	GTCTCGTGGGCTCGG
BRK81	GTCTCGTCCGCTCGG	CCGAAACAACTTCACAAGATGAGG	GTCTCGTGGGCTCGG
BRK82	GTTAGTTGATGTAGT	TAGTCCTGGAACTCGACATACCGT	GTCTCGTGGGCTCGG
BRK83	GTCTCGTCCGCTCGG	TTCGACCTTACCTAGATCAAGCCA	GTCTCGTGGGCTCGG
BRK84	GTTAGTTGATGTAGT	TGGCACAGGTTCTAGGTCCACTAC	GTCTCGTGGGCTCGG
BRK85	GTCTCGTCCGCTCGG	GATCATCCAACTAACTCCTCCGTT	GTCTCGTGGGCTCGG
BRK86	GTCTCGTCCGCTCGG	TACTTACGCTTGTTGGGATCACCT	GTCTCGTGGGCTCGG
BRK87	GTCTCGTCCGCTCGG	CCTCCCTAACAACAGGAGCATGTA	GTCTCGTGGGCTCGG
BRK88	GTTAGTTGATGTAGT	CTGCTTCGGATCGGTAGTAGAAGA	GTCTCGTGGGCTCGG
BRK89	GTCTCGTCCGCTCGG	CAACTAGCCAAACATTGATGCTGT	GTCTCGTGGGCTCGG
BRK90	GTTAGTTGATGTAGT	GCCTCAAACCGTACCCTCTACATC	GTCTCGTGGGCTCGG
BRK91	TACATTGATGCATGG	AGTAGCGTGAGTTCCTATGGAGCC	GTCTCGTGGGCTCGG
BRK92	GTCTCGTCCGCTCGG	GGTCCTGTATCTTTCCACTCACAA	GTCTCGTGGGCTCGG
BRK93	GTCTCGTCCGCTCGG	CCCAAGTCTGAAGTGATGGAAACT	GTCTCGTGGGCTCGG
BRK94	GTTAGTTGATGTAGT	GTAGGTGGCAGTTTGAGGACAATC	GTCTCGTGGGCTCGG
BRK95	GTCTCGTCCGCTCGG	AAGTCCATTCTTCTTCCAGACAGG	GTCTCGTGGGCTCGG
BRK96	TACATTGATGCATGG	ATGGTGGACTCTATGACCGTTCAG	GTCTCGTGGGCTCGG

Barcodes sequence for PCR_2 (5'-3' orientation)
